# Supplementary material for: A sister lineage of the Mycobacterium tuberculosis complex discovered in the African Great Lakes region
Source: Nat Commun. 2020 Jun 9;11:2917. doi: 10.1038/s41467-020-16626-6 (PMC7283319; doi:10.1038/s41467-020-16626-6)
Supplement: Supplementary file 1 — Supplementary Information [file 41467_2020_16626_MOESM1_ESM.pdf]

Supplementary Information

A sister lineage of the *Mycobacterium tuberculosis* complex discovered in the African Great Lakes region

By Ngabonziza, Loiseau, Marceau et al.

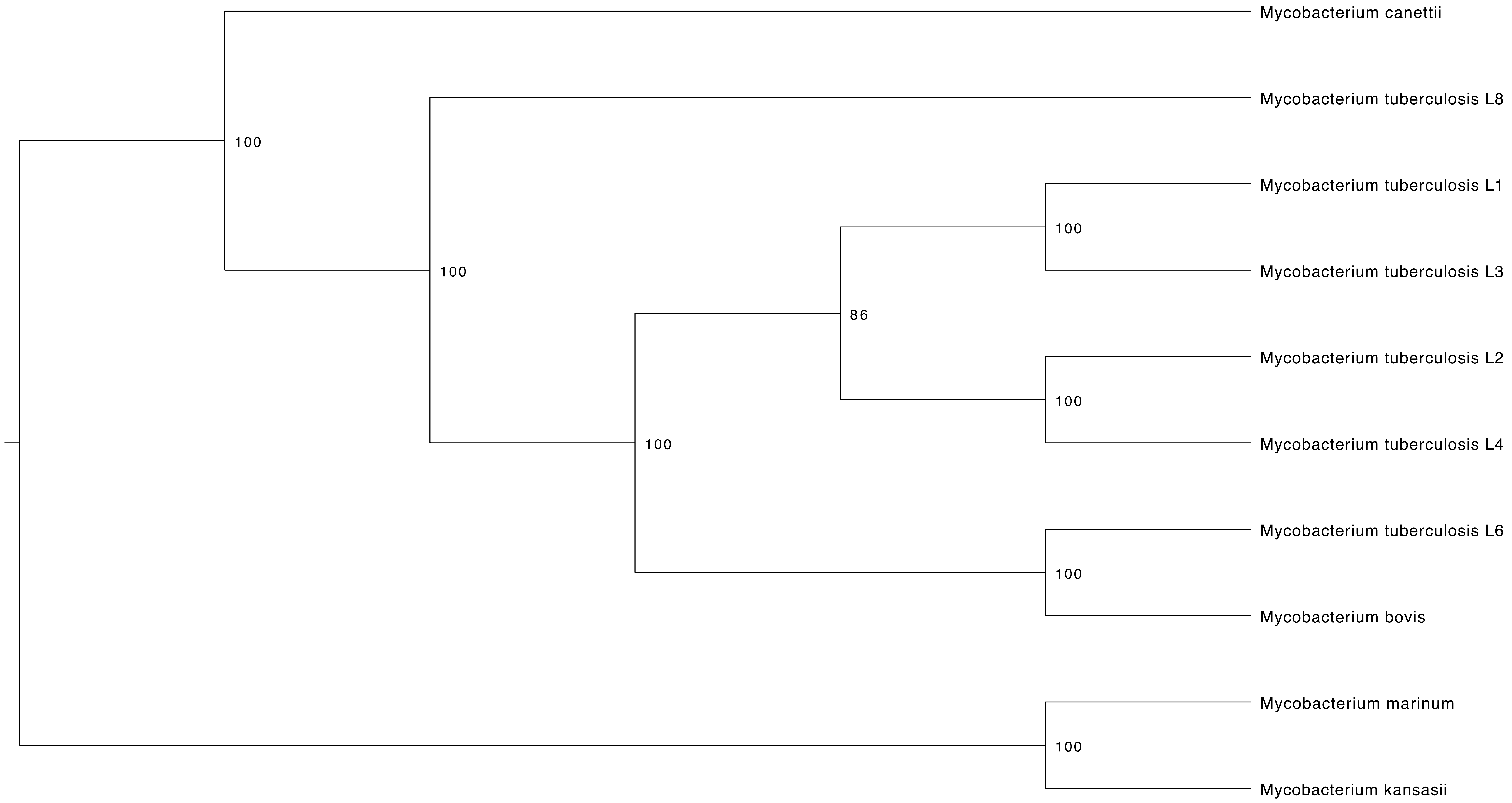

Supplementary figure 1: Core genome-based cladogram of the MTBC and outgroups. A phylogeny was constructed from the core alignment of L8, MTBC lineage representatives and *M. canettii* with *M. marinum* and *M. kansasii* as outgroups. Node labels represent bootstrap support for that split. Phylogeny is shown as a cladogram (branch lengths have no meaning) to allow clearer visualisation of the topology.

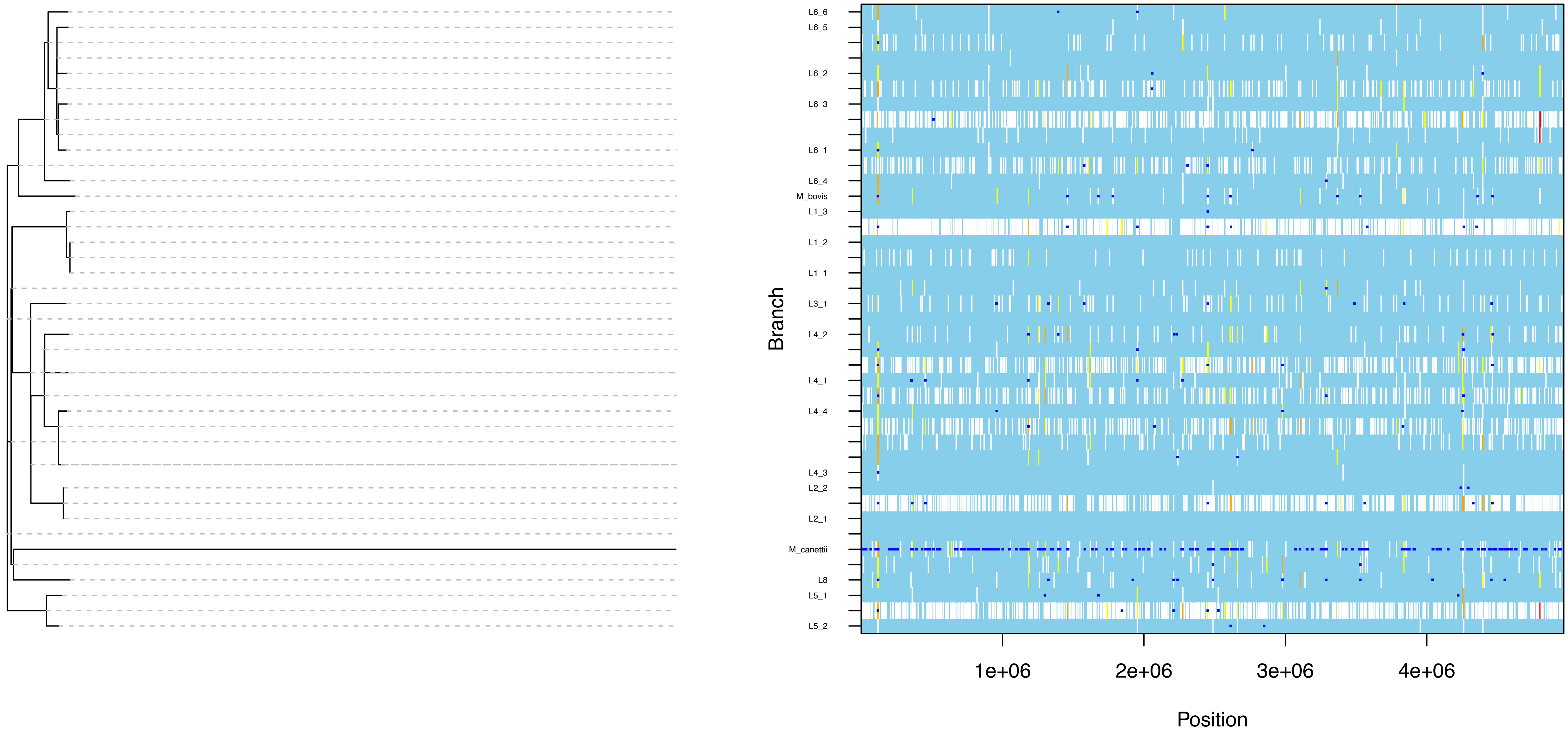

Supplementary figure 2: ClonalFrameML recombination analysis of the MTBC and *M. canettii*. White bars indicate reconstructed substitutions. Dark blue dots indicate areas of recombination.

Supplementary References

1. Cole, S. T., Brosch, R., Parkhill, J., Garnier, T., Churcher, C., Harris, D., Gordon, S. V., Eiglmeier, K., Gas, S., Barry, C. E., Tekaia, F., Badcock, K., Basham, D., Brown, D., Chillingworth, T., Connor, R., Davies, R., Devlin, K., Feltwell, T., Gentles, S., Hamlin, N., Holroyd, S., Hornsby, T., Jagels, K., Krogh, A., McLean, J., Moule, S., Murphy, L., Oliver, K., Osborne, J., Quail, M. A., Rajandream, M. A., Rogers, J., Rutter, S., Seeger, K., Skelton, J., Squares, R., Squares, S., Sulston, J. E., Taylor, K., Whitehead, S. & Barrell, B. G. Deciphering the biology of mycobacterium tuberculosis from the complete genome sequence. *Nature* vol. 393 537–544 (1998).
2. Zhu, L., Zhong, J., Jia, X., Liu, G., Kang, Y., Dong, M., Zhang, X., Li, Q., Yue, L., Li, C., Fu, J., Xiao, J., Yan, J., Zhang, B., Lei, M., Chen, S., Lv, L., Zhu, B., Huang, H. & Chen, F. Precision methylome characterization of Mycobacterium tuberculosis complex (MTBC) using PacBio single-molecule real-time (SMRT) technology. *Nucleic Acids Research* **44**, 730–743 (2016).
3. Zheng, H., Lu, L., Wang, B., Pu, S., Zhang, X., Zhu, G., Shi, W., Lu, Z., Wang, H., Wang, S., Zhao, G. & Zhang, Y. Genetic basis of virulence attenuation revealed by comparative genomic analysis of Mycobacterium tuberculosis strain H37Ra versus H37Rv. *PLoS ONE* **3**, (2008).
4. Miyoshi-Akiyam, T., Matsumura, K., Iwai, H., Funatogawa, K. & Kirikae, T. Complete annotated genome sequence of Mycobacterium tuberculosis Erdman. *Journal of Bacteriology* vol. 194 2770 (2012).
5. Loerger, T. R., Koo, S., No, E. G., Chen, X., Larsen, M. H., Jacobs, W. R., Pillay, M., Sturm, A. W. & Sacchettini, J. C. Genome analysis of multi- and extensively-drug-resistant tuberculosis from KwaZulu-Natal, South Africa. *PLoS ONE* **4**, (2009).
6. Mardassi, H., Namouchi, A., Haltiti, R., Zarrouk, M., Mhenni, B., Karboul, A., Khabouchi, N., Gey Van Pittius, N. C., Streicher, E. M., Rauzier, J., Gicquel, B. & Dellagi, K. Tuberculosis due to resistant Haarlem strain, Tunisia. *Emerging Infectious Diseases* **11**, 957–961 (2005).
7. Victor, T. C., De Haas, P. E. W., Jordaan, A. M., Van Der Spuy, G. D., Richardson, M., Van Soolingen, D., Van Helden, P. D. & Warren, R. Molecular Characteristics and Global Spread of Mycobacterium tuberculosis with a Western Cape F11 Genotype. *Journal of Clinical Microbiology* **42**, 769–772 (2004).
8. Fleischmann, R. D., Alland, D., Eisen, J. A., Carpenter, L., White, O., Peterson, J., DeBoy, R., Dodson, R., Gwinn, M., Haft, D., Hickey, E., Kolonay, J. F., Nelson, W. C., Umayam, L. A., Ermolaeva, M., Salzberg, S. L., Delcher, A., Utterback, T., Weidman, J., Khouri, H., Gill, J., Mikula, A., Bishai, W., Jacobs, W. R., Venter, J. C. & Fraser, C. M. Whole-genome comparison of Mycobacterium tuberculosis clinical and laboratory strains. *Journal of Bacteriology* **184**, 5479–5490 (2002).
9. Roetzer, A., Diel, R., Kohl, T. A., Rückert, C., Nübel, U., Blom, J., Wirth, T.,

Jaenicke, S., Schuback, S., Rüsch-Gerdes, S., Supply, P., Kalinowski, J. & Niemann, S. Whole Genome Sequencing versus Traditional Genotyping for Investigation of a Mycobacterium tuberculosis Outbreak: A Longitudinal Molecular Epidemiological Study. *PLoS Medicine* **10**, (2013).

10. Ilina, E. N., Shitikov, E. A., Ikryannikova, L. N., Alekseev, D. G., Kamashev, D. E., Malakhova, M. V., Parfenova, T. V., Afanas'ev, M. V., Ischenko, D. S., Bazaleev, N. A., Smirnova, T. G., Larionova, E. E., Chernousova, L. N., Beletsky, A. V., Mardanov, A. V., Ravin, N. V., Skryabin, K. G. & Govorun, V. M. Comparative Genomic Analysis of Mycobacterium tuberculosis Drug Resistant Strains from Russia. *PLoS ONE* **8**, (2013).
11. Miyoshi-Akiyama, T., Satou, K., Kato, M., Shiroma, A., Matsumura, K., Tamotsu, H., Iwai, H., Teruya, K., Funatogawa, K., Hirano, T. & Kirikae, T. Complete annotated genome sequence of Mycobacterium tuberculosis (Zopf) Lehmann and Neumann (ATCC35812) (Kurono). *Tuberculosis* **95**, 37–39 (2015).
12. Narayanan, S. & Deshpande, U. Whole-genome sequences of four clinical isolates of Mycobacterium tuberculosis from Tamil Nadu, south India. *Genome Announcements* **1**, (2013).
13. Tang, B., Wang, Q., Yang, M., Xie, F., Zhu, Y., Zhuo, Y., Wang, S., Gao, H., Ding, X., Zhang, L., Zhao, G. & Zheng, H. ContigScape: A Cytoscape plugin facilitating microbial genome gap closing. *BMC Genomics* **14**, (2013).
14. Wan, X., Qian, L., Hou, S., Drees, K. P., Foster, J. T. & Douglas, J. T. Complete genome sequences of Beijing and Manila family strains of Mycobacterium tuberculosis. *Genome Announcements* **2**, (2014).
15. Rodríguez, J. G., Pino, C., Tauch, A. & Murcia, M. I. Complete genome sequence of the clinical Beijing-like strain Mycobacterium tuberculosis 323 using the PacBio realtime sequencing platform. *Genome Announcements* **3**, (2016).
16. Chen, L., Zhang, D. T., Zhang, J., Su, Y. A. & Zhang, H. Whole-genome sequences of two clinical isolates of extensively drug-resistant Mycobacterium tuberculosis from Zunyi, China. *Genome Announcements* **2**, (2014).
17. Park, Y. K., Kang, H., Yoo, H., Lee, S. H., Roh, H., Kim, H. J. & Ryoo, S. Whole-genome sequence of Mycobacterium tuberculosis Korean strain KIT87190. *Genome Announcements* **2**, (2014).
18. Sung, W. R., Young, K. P., Park, S. N., Young, S. S., Liew, H., Kang, S. & Bai, G. H. Comparative proteomic analysis of virulent Korean Mycobacterium tuberculosis K-strain with other mycobacteria strain following infection of U-937 macrophage. *Journal of Microbiology* **45**, 268–271 (2007).
19. Rashdi, A. S. A. Al, Jadhav, B. L., Deshpande, T. & Deshpande, U. Whole-genome sequencing and annotation of a clinical isolate of Mycobacterium tuberculosis from Mumbai, India. *Genome Announcements* **2**, (2014).
20. Garnier, T., Eiglmeier, K., Camus, J. C., Medina, N., Mansoor, H., Pryor, M., Duthoy, S., Grondin, S., Lacroix, C., Monsempe, C., Simon, S., Harris, B., Atkin, R., Doggett, J., Mayes, R., Keating, L., Wheeler, P. R., Parkhill, J., Barrell, B. G., Cole, S. T., Gordon, S. V. & Hewinson, R. G. The complete genome sequence of Mycobacterium bovis. *Proceedings of the National Academy of Sciences of*

the United States of America **100**, 7877–7882 (2003).

21. Bentley, S. D., Comas, I., Bryant, J. M., Walker, D., Smith, N. H., Harris, S. R., Thurston, S., Gagneux, S., Wood, J., Antonio, M., Quail, M. A., Gehre, F., Adegbola, R. A., Parkhill, J. & de Jong, B. C. The genome of mycobacterium Africanum West African 2 reveals a lineage-specific locus and genome erosion common to the M. tuberculosis complex. *PLoS Neglected Tropical Diseases* **6**, (2012).
22. Supply, P., Marceau, M., Mangenot, S., Roche, D., Rouanet, C., Khanna, V., Majlessi, L., Criscuolo, A., Tap, J., Pawlik, A., Fiette, L., Orgeur, M., Fabre, M., Parmentier, C., Frigui, W., Simeone, R., Boritsch, E. C., Debrie, A. S., Willery, E., Walker, D., Quail, M. A., Ma, L., Bouchier, C., Salvignol, G., Sayes, F., Cascioferro, A., Seemann, T., Barbe, V., Locht, C., Gutierrez, M. C., Leclerc, C., Bentley, S. D., Stinear, T. P., Brisse, S., Médigue, C., Parkhill, J., Cruveiller, S. & Brosch, R. Genomic analysis of smooth tubercle bacilli provides insights into ancestry and pathoadaptation of Mycobacterium tuberculosis. *Nature Genetics* **45**, 172–179 (2013).
