# Supplementary material for: A sister lineage of the Mycobacterium tuberculosis complex discovered in the African Great Lakes region
Source: Nat Commun. 2020 Jun 9;11:2917. doi: 10.1038/s41467-020-16626-6 (PMC7283319; doi:10.1038/s41467-020-16626-6)
Supplement: Supplementary file 3 — Description of Additional Supplementary Files [file 41467_2020_16626_MOESM3_ESM.pdf]

Description of Additional Supplementary Files

File Name: Supplementary Data 1
Description: Genes absent in the MTBC lineage 8 genome and present in H37Rv and other MTBC genomes. Absent regions below 1kbp not considered. Absence/presence of the different IS6110-related genes is based on analysis of the local synteny of genes flanking the IS6110 element considered. The three major regions absent in lineage 8, also corresponding to known regions of difference (RD) among other MTBC genomes, are highlighted.

File Name: Supplementary Data 2
Description: Genes present in the complete MTBC lineage 8 genome and absent in H37Rv and/or other MTBC genomes. Regions below 1kbp not considered. Absence/presence of the different IS6110-related genes is based on analysis of the local synteny of genes flanking the IS6110 element considered. The complete lineage 8 genome from Rwanda contains a total of 21 IS6110 copies, versus 16 in H37Rv. The cobF region present in lineage 8 and M. canettii but absent in other MTBC genomes is highlighted.

File Name: Supplementary Data 3
Description: Gene scars detected in lineage 8, MTBC and M. canettii (STB) strains. ICDS, interrupted coding sequence; nt, nucleotide. The indicated ICDSs in H37Rv and MTBC genomes from other lineages than L8 correspond to intact coding sequence (CDS) both in lineage 8 and M. canettii (STB) strains as well as in M. marinum , indicating scars that likely occurred in the most recent common ancestor (MRCA) of all other MTBC lineages after its divergence from the common progenitor shared with lineage 8.

File Name: Supplementary Data 4
Description: Genes present in L8 and other MTBC genomes, and fully or partially absent from STB genomes.

File Name: Supplementary Data 5
Description: Supplementary 1. Information on the 38 complete genome sequences of MTBC and M. canettii strains used in this study (adapted from Yang; T., Zhong, J., Zhang, J., Cuidan, L., Yu, X., Xiao, J. et al. (2018). Pan-genomic study of Mycobacterium tuberculosis reflecting the primary/secondary genes, generality/individuality, and the interconversion through copy number variations. Front. Microbiol. 9. doi: 10.3389/fmicb.2018.01886).
