# Supplementary material for: A sister lineage of the Mycobacterium tuberculosis complex discovered in the African Great Lakes region
Source: Nat Commun. 2020 Jun 9;11:2917. doi: 10.1038/s41467-020-16626-6 (PMC7283319; doi:10.1038/s41467-020-16626-6)
Supplement: Supplementary file 9 — Reporting Summary [file 41467_2020_16626_MOESM9_ESM.pdf]

natureresearch

Corresponding author(s): NCOMMS-20-02690B

Last updated by author(s): YYYY-MM-DD

Reporting Summary

Nature Research wishes to improve the reproducibility of the work that we publish. This form provides structure for consistency and transparency in reporting. For further information on Nature Research policies, see Authors & Referees and the Editorial Policy Checklist .

Statistics

For all statistical analyses, confirm that the following items are present in the figure legend, table legend, main text, or Methods section.

n/a	Confirmed	
☒	☐	The exact sample size (*n*) for each experimental group/condition, given as a discrete number and unit of measurement
☒	☐	A statement on whether measurements were taken from distinct samples or whether the same sample was measured repeatedly
☒	☐	The statistical test(s) used AND whether they are one- or two-sided<br>*Only common tests should be described solely by name; describe more complex techniques in the Methods section.*
☒	☐	A description of all covariates tested
☒	☐	A description of any assumptions or corrections, such as tests of normality and adjustment for multiple comparisons
☒	☐	A full description of the statistical parameters including central tendency (e.g. means) or other basic estimates (e.g. regression coefficient) AND variation (e.g. standard deviation) or associated estimates of uncertainty (e.g. confidence intervals)
☒	☐	For null hypothesis testing, the test statistic (e.g. *F*, *t*, *r*) with confidence intervals, effect sizes, degrees of freedom and *P* value noted<br>*Give P values as exact values whenever suitable.*
☒	☐	For Bayesian analysis, information on the choice of priors and Markov chain Monte Carlo settings
☒	☐	For hierarchical and complex designs, identification of the appropriate level for tests and full reporting of outcomes
☒	☐	Estimates of effect sizes (e.g. Cohen's *d*, Pearson's *r*), indicating how they were calculated

Our web collection on statistics for biologists contains articles on many of the points above.

Software and code

Policy information about availability of computer code

Data collection	None
Data analysis	Genoscreen Deeplex-MycTB interface; Trimmomatic v0.33.22; SeqPrep; BWA v0.7.13; Picard v2.9.1; GATK v3.4.0; Samtools v1.2; VarScan v2.4.1; KvarQ; RAxML v.8.2.8; Canu v1.6; Circlator v1.5.5; pacbio-utils v0.2; snippy v4.4; BLAST; Artemis; MAGDA; Microscope platform v3.13.3; CheckM; MAUVE 2015-02-25; ClonalFrameML; Roary; RAxML-NG v 0.9.0;

For manuscripts utilizing custom algorithms or software that are central to the research but not yet described in published literature, software must be made available to editors/reviewers. We strongly encourage code deposition in a community repository (e.g. GitHub). See the Nature Research guidelines for submitting code & software for further information.

Data

Policy information about availability of data

All manuscripts must include a data availability statement. This statement should provide the following information, where applicable:

- Accession codes, unique identifiers, or web links for publicly available datasets
- A list of figures that have associated raw data
- A description of any restrictions on data availability

The complete genome sequence of the L8 strain from Rwanda was deposited in the NCBI repository under project PRJNA598991 with SRR10828835 and SRR10828834 accession codes for Illumina- and PacBio-derived genome sequences, respectively. The strain can be requested from the BCCM/ITM [http://bccm.belspo.be/] collection with accession code ITM-500961.

Field-specific reporting

Please select the one below that is the best fit for your research. If you are not sure, read the appropriate sections before making your selection.

- [x] Life sciences
- [ ] Behavioural & social sciences
- [ ] Ecological, evolutionary & environmental sciences

For a reference copy of the document with all sections, see nature.com/documents/nr-reporting-summary-flat.pdf

Life sciences study design

All studies must disclose on these points even when the disclosure is negative.

Sample size	All available strains for this lienage were included
Data exclusions	No data was excluded
Replication	Sequencing was the only experimental method and this does not require replication
Randomization	Experiment group comparisons were not done
Blinding	Blinding not required for comparative genomics study

Reporting for specific materials, systems and methods

We require information from authors about some types of materials, experimental systems and methods used in many studies. Here, indicate whether each material, system or method listed is relevant to your study. If you are not sure if a list item applies to your research, read the appropriate section before selecting a response.

Materials & experimental systems

n/a	Involved in the study	
x		Antibodies
x		Eukaryotic cell lines
x		Palaeontology
x		Animals and other organisms
	x	Human research participants
x		Clinical data

Methods

n/a	Involved in the study	
x		ChIP-seq
x		Flow cytometry
x		MRI-based neuroimaging

Human research participants

Policy information about studies involving human research participants

Population characteristics	A strain from a single patient was included. This patient was a male, aged 77 years, HIV-negative, resident of Rulindo district of Rwanda
Recruitment	The patient was recruited as MDR-TB patient into the DIAMA study. they gave informed consent for this and, after they passed away, their representative gave consent for the inclusion of identifying information. No biases are likely to affect the outcomes of this study.
Ethics oversight	The DIAMA trial was approved by the Rwanda National Ethical committee (IRB 00001487 of IORG0001100; Ref No. 0069/RNEC/2017)

Note that full information on the approval of the study protocol must also be provided in the manuscript.
